# Non-Intrusive Privacy-Preserving Approach for Presence Monitoring Based on WiFi Probe Requests

**DOI:** 10.3390/s23052588

**Published:** 2023-02-26

**Authors:** Aleš Simončič, Miha Mohorčič, Mihael Mohorčič, Andrej Hrovat

**Affiliations:** 1 Jožef Stefan Institute, Jamova 39, 1000 Ljubljana, Slovenia; 2 Jozef Stefan International Postgraduate School, Jamova 39, 1000 Ljubljana, Slovenia

**Keywords:** clustering, information element, MAC de-randomization, OPTICS, probe request, WiFi-enabled device detection, wireless sensing device

## Abstract

Monitoring the presence and movements of individuals or crowds in a given area can provide valuable insight into actual behavior patterns and hidden trends. Therefore, it is crucial in areas such as public safety, transportation, urban planning, disaster and crisis management, and mass events organization, both for the adoption of appropriate policies and measures and for the development of advanced services and applications. In this paper, we propose a non-intrusive privacy-preserving detection of people’s presence and movement patterns by tracking their carried WiFi-enabled personal devices, using the network management messages transmitted by these devices for their association with the available networks. However, due to privacy regulations, various randomization schemes have been implemented in network management messages to prevent easy discrimination between devices based on their addresses, sequence numbers of messages, data fields, and the amount of data contained in the messages. To this end, we proposed a novel de-randomization method that detects individual devices by grouping similar network management messages and corresponding radio channel characteristics using a novel clustering and matching procedure. The proposed method was first calibrated using a labeled publicly available dataset, which was validated by measurements in a controlled rural and a semi-controlled indoor environment, and finally tested in terms of scalability and accuracy in an uncontrolled crowded urban environment. The results show that the proposed de-randomization method is able to correctly detect more than 96% of the devices from the rural and indoor datasets when validated separately for each device. When the devices are grouped, the accuracy of the method decreases but is still above 70% for rural environments and 80% for indoor environments. The final verification of the non-intrusive, low-cost solution for analyzing the presence and movement patterns of people, which also provides information on clustered data that can be used to analyze the movements of individuals, in an urban environment confirmed the accuracy, scalability and robustness of the method. However, it also revealed some drawbacks in terms of exponential computational complexity and determination and fine-tuning of method parameters, which require further optimization and automation.

## 1. Introduction

Although presence and movement monitoring can have a negative connotation when applied to an individual, it also has the potential to improve and even save lives, but it must be conducted in accordance with some social consensus on preserving privacy. This challenge is being addressed with an increasing number of existing and emerging services and applications, for instance in the fields of public safety, transportation, urban planning, disaster and crisis management, mass events organization, etc. that depend on the information about people’s presence, proximity, occupancy, crowdedness, crowd dynamics, and movement patterns. Many of these services and applications relied on vision-based solutions until recently, when many camera-based monitoring systems have been shut down because they violated the new General Data Protection Regulation laws [1]. These systems are now being replaced by the development of new, less intrusive methods based on the indirect monitoring of devices equipped with various sensors and radio interfaces, e.g., WiFi, Bluetooth, 3G/4G/5G, etc.

As per development indicators collected by the World Bank, the number of cellular subscriptions in the world per 100 people grew from 87 in 2012 to 110 in 2021, reaching more than 100 in most developed countries, indicating that the mobile phone is the most omni-present personal device. In recent years, practically all smart mobile phones come with a number of wireless technologies including WiFi and Bluetooth in addition to supporting present and past generations of mobile network technologies (i.e., 2G–5G). For the association with available networks and provision of connectivity, all these technologies rely on exchanging some network management messages with network devices such as base stations and access points. Data in these messages can be used for non-intrusive detection of presence and movement patterns of individuals by way of tracking their carry-on devices. While there are strict legally enforced rules in place regarding the access to such data collected by mobile operators from mobile network technologies, wireless technologies have been more prone for potential misuse for instance due to the use of globally unique medium access control (MAC) addresses. Bluetooth relies on the short-range and point-to-point nature of its protocols to avoid tracking. Personal Bluetooth devices (e.g., mobile phones) also by default do not publicly advertise their presence. Wi-FI devices constantly scan and advertise their capabilities for better power management and quicker connection; therefore, they can be easily detected, recognized and tracked. This potential privacy breach led to the introduction of WiFi MAC address randomization, making the MAC address time-varying and random rather than globally unique. Recent randomization procedures in WiFi make it even more difficult to identify individual devices, as the sequence numbers of messages can also be randomized and the amount of data contained in messages can be reduced to only a few mandatory fields.

The recent COVID-19 pandemic with the introduction of preventive measures such as social distancing intensified investigations in non-intrusive privacy-preserving monitoring of individual’s social interactions largely by means of smart phones and various mobile apps (e.g., [2,3,4]). On the other hand, many applications still require only data on monitoring the presence and movement of anonymous individuals or crowds to provide an insight into actual patterns of behavior and enable the identification of hidden trends. In our study as part of the RESILOC project (https://www.resilocproject.eu/ (accessed on 22 February 2023)), we were focusing on the public safety domain, where knowing crowd densities, their dynamics and patterns in specific locations or strategic areas enables the planning and implementation of appropriate preventive measures and management strategies to improve the resilience of local communities. In particular, our goal was to develop a reliable system for the non-intrusive collection of anonymized information about the presence or movement patterns in terms of statistical counts without identifying and saving any privacy-sensitive information that would enable backtracking a device or an individual person, while efficiently avoiding possible double or multiple counts.

In this paper, we address the MAC address randomization problem for non-intrusive and privacy-preserving detection of WiFi-enabled devices. We propose a new MAC de-randomization method, which is able to recognize unique devices with high accuracy by grouping similar network management messages (called Probe Requests, PRs) based on the data in the message and the corresponding radio channel characteristics. The method uses a novel clustering and matching procedure that was calibrated with a publicly available labeled dataset. It was validated by the measurements performed using a custom-designed, low-cost system for capturing, transferring and storing WiFi PRs. The validation measurements took place in a fully controlled rural environment and a semi-controlled indoor environment at the Jozef Stefan Institute (JSI). An additional verification of the method in terms of scalability and accuracy was performed in a real, crowded urban environment in the city of Catania with high-density pedestrian traffic and consequently a high number of devices transmitting a large number of PRs. The main contributions of this work are:

1. The design and implementation of a low-cost system for capturing, transferring and storing WiFi PRs and corresponding radio channel characteristics.
2. Open datasets of the captured WiFi PRs and corresponding radio channel characteristics in a controlled rural outdoor, semi-controlled indoor and uncontrolled urban outdoor environments.
3. A novel MAC de-randomization method for distinguishing individual WiFi-capable devices including new clustering and matching procedures based on PRs and corresponding radio channel characteristics.
4. Validation of the proposed method by the measurements in controlled, semi-controlled and completely uncontrolled environments.

The rest of the paper is organized as follows. Section 2 provides the necessary background on the structure of WiFi PRs and randomization of MAC addresses, and it outlines the related work on WiFi based monitoring of population behavior. Section 3 describes the proposed system architecture and its implementation using wireless sensing devices (WSDs) and a remote server with database for storing raw data. Section 4 gives a detailed description of the proposed MAC de-randomization method including the procedures for the collection and pre-processing of data as well as for the clustering and matching of PRs for identifying unique WiFi-enabled devices. Section 5 defines the validation scenarios considering different numbers and groupings of devices and different operating environments before it provides performance evaluation results. Finally, Section 6 concludes the paper and outlines some ideas for future work.

## 2. Background and Related Work

Exploiting the information acquired from the WiFi network management messages has been a widely used approach for user tracking, crowd monitoring, presence detection, etc. This approach has become quite challenging in recent years, which is mainly due to the privacy guarantees that manufacturers achieve by randomizing the MAC addresses of WiFi interfaces. Therefore, device-based passive tracking approaches that detect a device carried by a user had to address the issue by new methods that consider several additional parameters/data extracted from the WiFi management frames. In the following, we briefly present the basic types of WiFi management frames that contain data useful for methods to determine the number of devices or to distinguish between individual detected WiFi-enabled devices and MAC addressing randomization followed by a brief overview of related research and applications.

### 2.1. Probe Requests and MAC Randomization

The basic idea of non-intrusive presence monitoring is to exploit the standard operation of WiFi-enabled devices, i.e., their activity when not connected to an operational WiFi network. In general, WiFi technology is based on the 802.11 standard that defines several frame types which are categorized in three major groups, namely, (i) data frames for data transmission, (ii) control frames for controlling the access to the wireless medium and for validation of received frames, and (iii) management frames used by supervisory functions (e.g., associating the device with the network, roaming between access points, etc.). When a device with an enabled WiFi interface is in an unassociated state, i.e., not connected to a network, it carries out passive and/or active scanning for available WiFi networks that it could connect to. Both approaches rely on WiFi management frames which are not encrypted as they do not contain any user data. Passive scanning refers to devices that are waiting and listening for announcements from access points sent in beacon frames, successively moving through the entire set of channels. Active scanning relies on WiFi-enabled devices sending PR frames on a selected channel and waiting for a probe response from nearby access points, and if no response is received for a certain time, repeating the procedure on the next channel.

**Figure 1:**
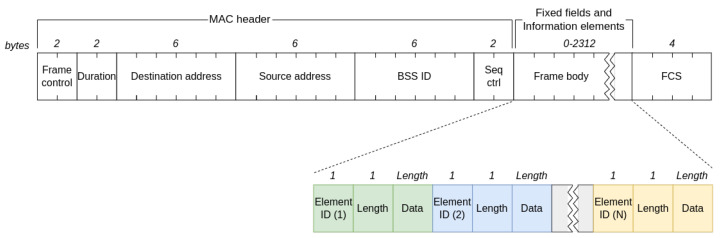
Generic 802.11 management frame with IE fields in the frame body.

Figure 1 shows a generic WiFi management frame. Management frames are denoted by the *Subtype* field of the Frame Control field. Frame body of management frames uses fixed-length fields called *fixed fields* and variable-length fields called *Information Elements (IE)*. While fixed fields do not have a header as their length and order are predefined, the first octet in IE fields defines the element ID and the second octet defines its length. The mandatory IEs in the PRs’ body frame are Service Set Identifiers (SSIDs), i.e., the unique names of WiFi networks that the device was already associated with in the past, and supported data rates. Based on IE data in the received PR, the access point determines whether the device fulfills the conditions to join the network or not.

By actively scanning for available WiFi networks, a WiFi-enabled device becomes discoverable to nearby listeners since it needs to include its MAC address in the source address field of PR. In case of having a globally unique MAC address, this makes it distinguishable from other devices in the network. MAC addresses have a standardized length of six octets. In a globally unique MAC address, the first three octets, called an Organization Unique Identifier (OUI), are unique to a manufacturer and are defined by IEEE. The last three octets, called the Network Interface Controller (NIC), are assigned by a device manufacturer to make each device uniquely distinguishable.

Since the MAC address is tied to a device and consequently to the person carrying it, manufacturers started to implement different schemes to randomize MAC adressess and thus protect user privacy. The seventh bit in the first octet of the MAC address indicates whether a device is using a globally unique address that is constant over time (bit set to ‘0’) or a randomized address (bit set to ‘1’). Apple with iOS version 8.0 was the first to release its devices with MAC randomization for mass sale in 2014. Although MAC randomization is now implemented by almost every device for pre-association state and for post-association state with an access point [5], the process of randomization is not standardized. Some manufacturers randomize the entire MAC address, while others use a fixed Company Identifier (CID) for first three octets and randomize the remaining three octets. To prevent tracking a particular MAC address, devices are changing their MAC address over time. The frequency or specific time events of generating new MAC addresses are also not standardized. Some devices change their MAC address for each burst that PRs are sent, while others change it less frequently. The entire process of generating and changing MAC addresses is managed by the operating system.

Undocumented and proprietary source codes make it difficult to analyze vulnerabilities in the randomization process. However, previous works show that tracking WiFi-enabled devices is possible using various techniques that differ in terms of the frame type, state of communication, physical characteristics of the radio waves, radio channel and transceiver characteristics of the particular device [5]. Some techniques, such as using the WPS field in PR to infer the actual MAC address of the device or using the sequence number to distinguish mobile devices, are already outdated, which shows the efforts of manufacturers to quickly fix the potential privacy vulnerabilities. To make it even more challenging to identify a unique WiFi device, some manufacturers have introduced sending subsequent PRs that contain different data, which makes it difficult for the observer to associate them to a single device, or they send PRs that contain only the mandatory IEs. In this way, the data from different mobile devices look very similar and are therefore harder to distinguish.

### 2.2. Related Work

Numerous solutions have been proposed in the literature for monitoring population behavior in terms of crowdedness, density, presence, proximity, etc., based on WiFi traffic analysis. Early solutions were based on simply tracking MAC addresses, assuming that MAC addresses are unique for each WiFi-enabled device [6]. These solutions are now outdated due to MAC address randomization. The privacy became important in any system that collects data and in particular if it processes user-related information, as data protection rights under the GDPR (e.g., Regulation EU 2016/679) must be fully met. In recent years, MAC address randomization has attracted a lot of attention from vendors, who have developed and implemented various solutions [7]. An extensive test of different solutions was carried out [5] to determine the usage of randomization, under what conditions MAC address randomization is performed, and if the tracking vulnerabilities are suitably mitigated.

To distinguish between different WiFi-enabled devices while protecting user privacy, different approaches and techniques have been applied. Initially, differentiating between unique devices was based on the timing analysis of PR frames. In [8], the authors measured the time between received PRs on different channels. Since the time delay is not specified by the standard and depends on the configuration of a device, it is a suitable feature for fingerprinting. A similar approach based on timing analyses of the PR frames is reported in [9]. The de-randomization of MAC addresses can be based on timing attacks, where the inter-frame arrival times of PRs are used to group frames coming from the same device, although they use distinct MAC addresses as proposed in [10]. Frames are grouped by several distance metrics based on the timing and incremental learning algorithm.

Timing as a distinction feature is unreliable in real-world environments due to scattering and multi-path phenomena which introduce some random delays between probes and bursts [11]. Thus, more reliable solutions to fingerprint the devices based on IEs in PR have been widely addressed in the literature. In [12], a study of IEs is presented, and new fields and techniques to track users are identified. It was demonstrated that scrambler seeds of commodity WiFi radios are predictable and can be used for device identification. In addition, two attacks that reveal the real MAC address of devices were presented.

Many applications for counting people at a specific location based on PRs were developed as in [13], where the goal was to count the passengers in public transport. It is worth noting that the majority of Android handsets did not use randomized techniques in that study. A solution for counting participants in public demonstrations proposed in [14] was based on a WiFi PR broadcast by the phones. The basic signal behavior was investigated by applying a distance filter based on RSSI, which is impractical due to applying a common threshold, and time-based filters, which have extra requirements regarding the scanner setup and increase the likelihood that counted devices actually belong to participants. Results showed that the count from analyzing PRs represented only a small fraction of the actual attendance. A method for pedestrian counting, classifying them as moving or static and locating them in a road intersection, is exploited in [15] and compared with machine learning (ML) techniques in [16]. It is based on power measurements and PRs; however, it does not provide a description of the MAC randomization procedure used. The solution for understanding the behavior of visitors in [17] addresses the issue of the device randomization by using more than 1.7 million PR frames, historical transition probability and a Hidden Markov Model (HMM)-based trajectory inference algorithm. The group behavior detection system introduced in [18] is also based on capturing PRs and tackling MAC address randomization by the method described in [7] based on collective matrix factorization, which reveals the hidden associations by factorizing mobility information and usage patterns simultaneously.

In [19], an approach for estimating the presence of mobile devices at a certain place in time is proposed, which is immune to MAC address randomization. The approach is based on the state machine to detect the arrival, presence and departure of devices in the sensor proximity. A novel architecture for people mobility monitoring and analyzing a solution based on PRs is presented in [20]. The main features include the preservation of user privacy, extraction of key metrics on user return and permanence, and computation of mobility heat maps. Another solution for studying the mobility patterns proposed in [21] is based on PRs which correlate the multi-dimensional statistical properties of the captured PRs split by brands with the actual ground truth, which is manually labeled for creating a regression model. A low-cost, high-reliability and low-complexity real-time passenger counting system is reported in [22] with a highly accurate mathematical model in conjunction with the MAC data provided by the developed system and applied Kalman filter. The authors in [23] have developed a crowd estimation scheme that addresses MAC randomization by exploiting the fact that the number of PRs in a defined time interval changes proportionally to the number of devices present. The conversion factor between the number of devices and the number of PRs was determined by measuring the bursts per minute of PRs from 75 devices, whereas the authors in [24] used a statistical approach to estimate the client population from PR counts without requiring an additional ground truth technique.

Vision and TrueSight crowd monitoring algorithms that estimate the number of devices were exploited in [25] to prove that despite MAC address randomization, MAC address-based crowd monitoring is still a viable solution. Both approaches mitigate the influence of the randomization by PRs. While Vision uses data and beacon packets, TrueSight uses PR sequence numbers and hierarchical clustering. A similar approach of tracking the sequence number of the PR, assuming that the number increases with newly received PR, is given in [26], which also incorporates the timestamp of the received PR. Based on these parameters, a de-randomization algorithm calculates the score for each couple of random MAC addresses and identifies which MAC addresses belong to the same device based on the defined threshold. An algorithm incorporated in the system for analyzing urban mobility is applied in [27] for the privacy-preserving detection of people’s flow by an ML approach [27] and in [28] where two methodologies for people counting and mobility detection are proposed. However, new privacy-preserving approaches tend to completely randomize the sequence number of PR which makes the previously mentioned methods unreliable [5]. Despite the enhanced randomization, the authors in [29] developed an efficient crowd monitoring system based on the passive detection of PRs. The algorithm counts the devices from a measured rate of PR burst (PRs received in 10 ms) transmissions using a statistical estimator. Another solution for crowd monitoring based on the information in PR is proposed in [30], where the SSID information of the preferred WiFi access points included in PR is exploited in post-processing together with the information of the existing WiFi access points to determine the daily number of visitors at different locations.

A solution for estimating the number of people proposed in [31] is based on a footprint mechanism to overcome MAC address randomization, generating an identifier for probes based on MAC addresses and other IE data. The test in real scenarios showed that the solution can achieve the accuracy close to 95%. Based on the analyses of the influence that different MAC address randomization schemes have on statistical counts of the WiFi-based monitoring systems, another approach was proposed and verified in [1], which is based on the DBSCAN clustering algorithm and two fingerprinting features. The first feature consists of an ordered list of IEs and certain bitmasks available in PR, while the second feature is based on the burst length and the arrival time difference between PRs within that burst, or the Inter-Frame Arrival Time (IFAT).

To estimate the number of WiFi-enabled devices in a given area, the authors in [11] also used clustering algorithms, namely DBSCAN and OPTICS. They extracted the most relevant IEs from PRs and used their data lengths as features to distinguish between different devices. Additionally, they wrote an algorithm to dynamically detect which IEs are changing between different PRs and use only the most relevant ones. In laboratory testing, they achieved 91.3% accuracy with the OPTICS algorithm. The authors extended their work in [32], where they also considered pseudo-random MAC address detection, HDBSCAN as a clustering algorithm, and additional features for burst identification: namely, sequence number, RSSI, and time of arrival. In a controlled environment, they achieved 96% accuracy, while in the real scenario, they achieved an average accuracy of 75%. Since PRs from two different devices can contain IEs of the same lengths, this method is prone to undercounting the number of unique WiFi devices. The authors in [33] also used the DBSCAN algorithm and a publicly available dataset [34] for clustering PRs. They determined the impact of IE as features for the clustering algorithm by computing Gini importance with Random Forests, and by using the most important features, they achieved a clustering accuracy of 92%. Since the approaches applying the DBSCAN and OPTICS algorithms gave the most promising results in the related literature, they were also used as the basis for developing the algorithm described in this paper.

## 3. System Design, Implementation and Deployment

**Figure 2:**
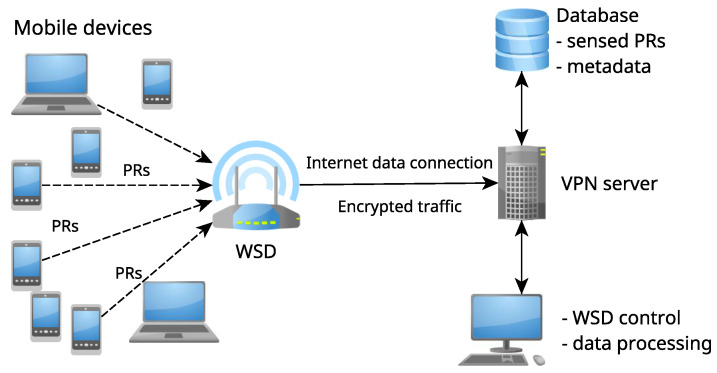
Schematic of system design and flow of data.

Leveraging the existing off-the-shelf technologies, a low-cost system shown in Figure 2 was designed and built to monitor and capture WiFi network management messages. These messages are captured passively (without user or device interaction) by listening for PR packets on a single channel of the 2.4 GHz WiFi band.

### 3.1. System Architecture

The system shown in Figure 2 is composed of a WSD for capturing WiFi network management traffic which is connected either via the wireless access point or with an Ethernet cable through a secure VPN connection to the remote database. The database stores raw data for further processing, together with additional metadata about the deployment and optional pre-processed data. Once the data are stored in the remote database, it can be further analyzed either in real time or by using more complex algorithms for post-processing the collected data.

### 3.2. Capturing the WiFi Network Management Traffic

Passive monitoring of the network management traffic is performed by a WSD listening to the WiFi traffic in a 2.4 GHz band and filtering out the PR packets. On a WSD, certain minimum computing power is required for filtering, pre-processing, and transmitting the collected data. This dictates the minimum capabilities of CPU and connectivity options and limits the software stack used.

Due to its relatively low cost, good support and known capabilities, the Raspberry Pi (rPi) device (version 4 with 2 GB of internal memory) was selected to be used as the WSD. The rPi’s built-in WiFi adapter and drivers do not support the operation in a so-called monitor mode required for capturing the WiFi traffic. Therefore, the built-in adapter is used as a default connection to the Internet for the transfer of captured data to the database, whereas an additional USB dongle using a Realtek RTL88212au chipset with open-source drivers was used to enable the monitor mode of operation. In addition to capturing WiFi PR packets, the USB dongle on the WSD is also capable of capturing Bluetooth beacon advertisements from Bluetoth tags or devices if enabled.

The operating system used for the WSD is a minimal installation of 64 bit Raspberry Pi OS. A custom service is run upon the device startup to automatically start wireless data collection. The traffic monitoring data are collected using the Tshark packet capture program, filtered and parsed in Python using the PyShark package. The collected data are transferred in a JSON format to the PostgreSQL database via HTTPS REST (Representational State Transfer) protocol calls to the remote server through a secure VPN connection, ensuring that the sensitive data are not exposed at any point in the communication pipeline. The described data flow is depicted in Figure 2.

The database used to store raw data collected from WSDs is an instance of the PostgreSQL database running on remote infrastructure. The collected raw data are stored together with the corresponding metadata. Periodically, every ten seconds, the WSD transmits collected data to the database. Each PR contains all of the information from the captured packet in a verbose, human readable JSON format.

### 3.3. System Deployment

Before the system is deployed in the targeted operating environment, WSDs are pre-programmed, pre-installed and tested in a controlled laboratory environment. When a device is set up and turned on in the field, it automatically creates an access point for the user who connects to the access point and sets the metadata (e.g., location and ID of the device). The connection to the Internet can be established either via another access point or via the Ethernet cable. The system was deployed at multiple locations as described in Section 5.1, each with specific observable variations in WiFi traffic.

## 4. Detecting Unique WiFi Interfaces

To determine the actual number of WiFi-enabled devices at a certain location covered by WSD in a completely anonymous and unobtrusive way, we developed and implemented a method for the de-randomization of MAC addresses of the WiFi interfaces integrated in devices. In this respect, we exploit the data collected by WSDs which can be, after the processing phase, further used for different use cases, namely (i) defining the number of people at a location of interest in given time or in different time frames; (ii) analyzing population movement patterns in streets, shopping malls, etc. in different timeframes; (iii) counting crossings or passages over bridges, entrances, streets, etc.; (iv) real-time adaptations of emergency exits/directions; etc.

### 4.1. Data Collection

The authors in [11,32] used only the length of the data from the IE fields in PR to de-randomize MAC addresses of WiFi-enabled devices and identify their number in a given area. In this study, we adopted a similar approach as in [34] and also considered the data itself, along with time of arrival (ToA) and RSSI, to reduce the problem of undercounting. With the collected information, two different WiFi-enabled devices can be distinguished, or a WiFi-enabled device sending PRs with different MAC addresses can be detected. The proposed procedure takes into account that data in PRs are strongly influenced by the chipset, the device driver and the WiFi software stack.

The first step in collecting the information regarding PRs was to identify which IEs and other PR information are more specifically characterizing a given device. In [12,35], the authors calculated the entropy and stability of IEs in datasets and investigated which IEs are changing between devices and which IEs are stable for a particular device. The goal was to choose IEs with high entropy, meaning that the data from IEs for different devices are considerably different, and at the same time highly stable, so that the data from IEs for a particular device are stable over time and do not estimate a single device as multiple devices.

Based on this, we decided to collect from each received PR the information about MAC address, *Supported Data Rates*, *Extended Supported Rates*, *HT Capabilities*, *Extended Capabilities*, *Interworking*, *VHT Capabilities*, data under *Extended Tag* and *Vendor-Specific Tag*, *RSSI*, *SSID* and the timestamp when PR was received. Note that not every PR includes all the parameters specified above, as all IE fields except *Supported Data Rates* and *SSID* are optional, so the information about which IEs a given WiFi-enabled device is transmitting is also relevant in device characterization. Selected IEs with information about the stored data type and data length are listed in Table 1. *Supported Data Rates* and *Extended Supported Rates* are represented as arrays of values that encode information about the rates supported by a mobile device. The rest of the data from IEs is represented in hexadecimal format. The *Vendor-Specific Tag* is structured differently than the other IEs. This field can contain multiple vendor IDs with multiple data IDs and corresponding data. Similarly, the *Extended Tag* can contain multiple data IDs with corresponding data.

### 4.2. Data Pre-Processing and Storing

**Figure 3:**
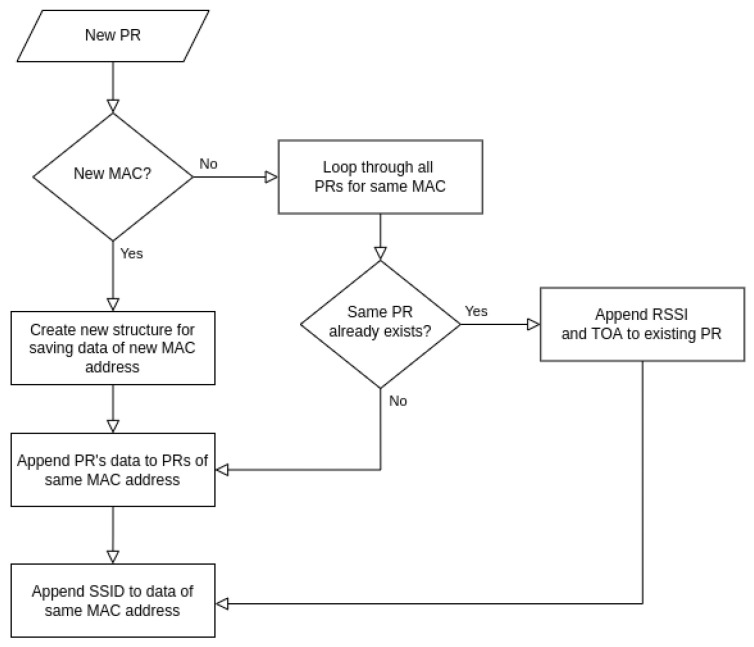
Algorithm for saving data from received new PRs.

WSDs scan for PRs for a predefined scan time, and during this time, the data from IE fields are pre-processed and saved in a predefined JSON structure before being transferred to the database. For more information about the structure of saved data, see Appendix A. The pre-processing procedure is depicted graphically in Figure 3.

For each new PR, the procedure first checks if its MAC address has already been detected and saved in the current scan time. If not, a new data structure is created under the new MAC address for storing PR’s IE data and SSIDs. If the new PR contains one of the already existing MAC addresses from the current scan time, the procedure compares new IE data with already recorded IE data for the same MAC address. If identical PR’s IE data from the same MAC address are already stored, then only data for *ToA*, *RSSI* and *SSID* are appended to the existing data structure. Thus, the database is reduced and PRs can be compared more efficiently. However, if no identical PR’s IE data have yet been recorded with the same MAC address, then a new data structure under the same MAC address with new PR’s IE data and possible new SSIDs is appended.

At the end of each scan time, all processed data are sent to the database along with additional metadata about the collected data such as WSD serial number and scan *start time* and *stop time*.

### 4.3. De-Randomization Method

To estimate the number of unique WiFi devices during a selected time interval in the area covered by a specific WSD, the de-randomization method of gathered PRs has to be performed. This can be accomplished by clustering PRs with respect to their similarity. The data from different MAC addresses are compared by calculating their *distance* whereby MAC addresses with very similar data have small distance and vice versa.

The proposed method for matching MAC addresses comprises the following steps:

1. MAC addresses are first divided into two groups: global and random addresses. Additionally, random MAC addresses are also subgrouped with respect to the CID part of the MAC address.
2. The clustering of random MAC addresses is applied to all groups with random MAC addresses to obtain clusters from individual WiFi-enabled devices.
3. The clustering of global addresses with clusters of random addresses is applied to match global MAC addresses with clusters of random MAC addresses obtained in the previous step.
4. The number of individual WiFi-enabled devices is estimated by counting the number of clusters.

#### 4.3.1. Initial Grouping of MAC Addresses

Since the data for each scan interval are sent to the database in packets, these packets are merged to match the desired time interval in which the de-randomization method is performed. In addition, the packets are filtered based on the serial number of the WSD that sent data to the database so that only data from one WSD is processed at any given time. Next, the algorithm for merging PRs from the same MAC address is applied, similar as used in the first pre-processing phase. In addition, each MAC address and its data are placed into a global or random group based on the *local* bit in the MAC address.

**Figure 4:**
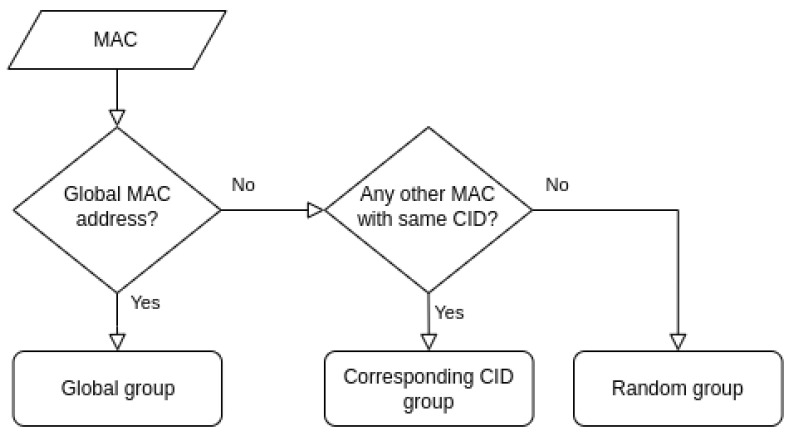
Algorithm for the initial grouping of MAC addresses and corresponding data.

Furthermore, random MAC addresses are mapped according to their CID value. If no *CID group* is found for a particular MAC address, it is assigned to a *Random group* which corresponds to devices with random MAC address and no manufacturer identifier. This additional subgrouping allows for the finer matching of similar MAC addresses, as it is assumed that a WiFi-enabled device that sends PRs with a completely random MAC address will not send subsequent PRs with a known CID as part of its MAC address. The described initial grouping procedure is shown in Figure 4.

#### 4.3.2. Clustering of Random MAC Addresses

The clustering of random MAC addresses starts with the calculation of the distance matrix for each *CID group* and for the *Random group*. In particular, the distance between all pairs of MAC addresses is calculated and stored in a 2D array. The distance depends on the similarity of the PR’s IE fields. Each IE is assigned one or more coefficients that reflect its weight. IEs that vary considerably between different WiFi-enabled devices and also have high stability for the same WiFi-enabled device have a higher weight and vice versa. For example, the distance decreases for each *SSID* that the two PRs have in common, while it increases for each supported data rate that they do not have in common. The distance is also affected by *RSSI* (increased when the absolute difference exists) and *ToA* (decreased when the absolute difference is less than a certain threshold). For other fields considered (i.e., *HT Capabilities*, *Extended Capabilities*, *Extended Tag*, *Vendor-Specific Tag*, *Interworking* and *VHT Capabilities*), the distance is increased proportionally to the number of different bits. The pseudocode for calculating the distance between two PRs can be found in Appendix B as Algorithm A1.

The distance for each PR from one MAC address (N) to each PR of another MAC address (M) is calculated. Then, the distance for these two MAC addresses is defined as an average of one-third of the shortest distances, which is inserted in an NxM distance matrix.

The distance matrix of random MAC addresses represents an input to a density-based clustering OPTICS (Ordering Points to Identify the Clustering Structure) algorithm [36]. In the proposed approach, we used a specific implementation of the OPTICS algorithm from the scikit-learn library (https://scikit-learn.org/stable/ (accessed on 22 February 2023)). The algorithm orders the data points so that the spatially closest points become neighbors. A point is classified as a core point if at least *MinPts* points are found within its neighborhood with a predefined radius. To detect a change in the density of points, the OPTICS algorithm defines two additional parameters for each point: the core distance and the reachability distance. The core distance is undefined if a point is not a core point; otherwise, it is equal to the minimum value of the neighborhood radius required to classify a given point as a core point. The reachability distance is defined for a selected point in relation to another point. It is the maximum value of the distance between these two points or the core distance of a point. If the selected point is not a core point, the reachability distance is set to undefined.

**Figure 5:**
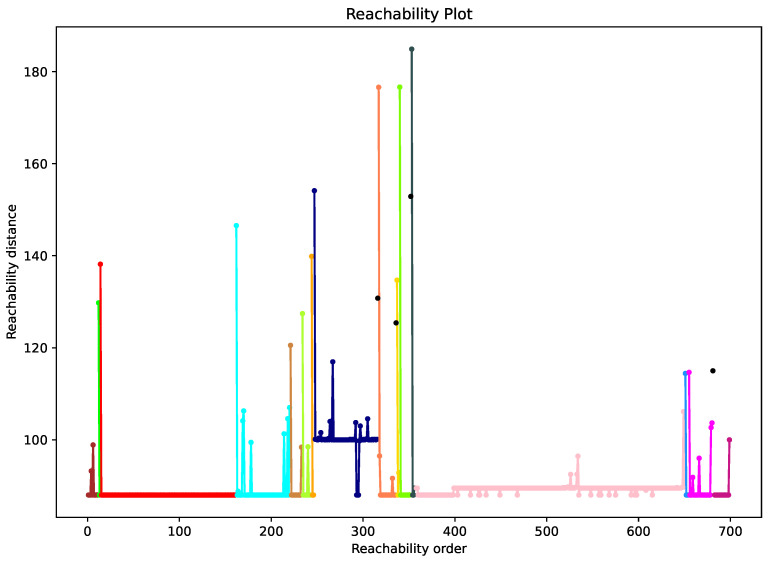
Reachability plot for data collected at the city square Piazza Università in Catania in an early morning 15-min interval. Points of the same cluster have the same color.

The OPTICS algorithm does not explicitly cluster the data into groups. Its output is a visualization of the reachability distances of points in the same order as processed by the algorithm. The order in which the algorithm selects points is based on the reachability distances. The point with the smallest reachability distance is selected first. The resulting 2D representation is called a reachability graph and is shown in Figure 5. The points belonging to the same cluster have low reachability distance, so they appear as valleys on the reachability diagram. The valleys are separated by spikes corresponding to the distances between clusters or between a cluster and a noise point. In other words, the peaks on the reachability plot indicate the beginning of a new cluster, as also indicated in Figure 5 by different colors.

In the last step, the reachability distances obtained by the OPTICS algorithm are used in a new optimized algorithm for clustering random MAC addresses, as provided in Appendix B as Algorithm A2. The main principle of the algorithm is to detect the point in the reachability distances at which the curve starts to drop. If the drop is larger than the predefined threshold, the subsequent points are grouped in a new cluster until the values start to increase and exceed the predefined threshold, indicating the end of the cluster.

The described algorithms are applied to each *CID group* and to the *Random group*. As a result, clusters of MAC addresses that likely correspond to the same WiFi-enabled device within each *CID group* and the *Random group* are obtained. The data from MAC addresses clustered together are merged and used later for matching with global MAC addresses.

#### 4.3.3. Matching of Global MAC Addresses with Clusters of Random MAC Addresses

The next step of the de-randomization method matches the global MAC addresses with already clustered random MAC addresses. The distance matrix is first calculated between the data of each global MAC address and the data of each cluster of random MAC addresses, whereby the distances between the global MAC addresses and the distances between the clusters of random MAC addresses are set to infinity to prevent matching.

Then, the matching algorithm iterates through the global MAC addresses and checks the distances to the clusters of random MAC addresses. If the smallest distance is less than the specified threshold and the next smallest distance is larger for a predefined factor, then the cluster of random MAC addresses with the smallest distance is a good candidate for matching with the global MAC address. In addition, all the distances for the selected cluster of random MAC addresses are checked. If the smallest distance corresponds to the same global MAC address and the next smallest distance to other global MAC address is larger by a certain factor, the global MAC address is matched to the cluster with random addresses, and their data are merged.

The code for the data pre-processing and the de-randomization method was written in the Pyhton programming language and is freely available to allow replication and continuation of the work (https://gitlab.com/e62Lab/resiloc_project/wireless-data-analysis (accessed on 22 February 2023)).

## 5. Performance Evaluation and Discussion

The proposed MAC de-randomization method has been tested and validated on datasets collected in different operating environments with different WiFi-enabled devices and with different test scenarios, including labeled datasets, datasets from a controlled and a semi-controlled environment, and a dataset from a challenging, completely uncontrolled environment to test robustness and scalability.

### 5.1. Testing Scenarios and Methodology

The datasets for performance evaluation of the proposed MAC de-randomization approach include the publicly available labeled dataset [34] as well as datasets from the measurements in three different environments, namely (i) a controlled rural environment, (ii) a semi-controlled indoor environment, and (iii) an uncontrolled urban environment, as described in the following. Measurements in rural and indoor environments were carried out under different testing scenarios, while no specific scenarios could be implemented in the uncontrolled urban environment, where only the robustness and scalability of the proposed approach could be tested under high density of WiFi-enabled devices.

For the initial testing and setting the parameters of the MAC de-randomization method, we used the publicly available labeled dataset [34] obtained with 22 devices among which 18 used MAC randomization. It contains 20-min captures of PRs in three non-overlapping WiFi channels (1, 6, and 11) for each individual device in six different operating modes (i.e., combinations of settings based on display status, Wi-Fi connectivity, and power saving) saved in 315 .pcap files. Data collection was performed in an anechoic chamber and in pseudo-isolated environments. In pseudo-isolated environments, additional filtering was performed based on the MAC address of known nearby devices and based on the power threshold of unknown nearby devices.

**Figure 6:**
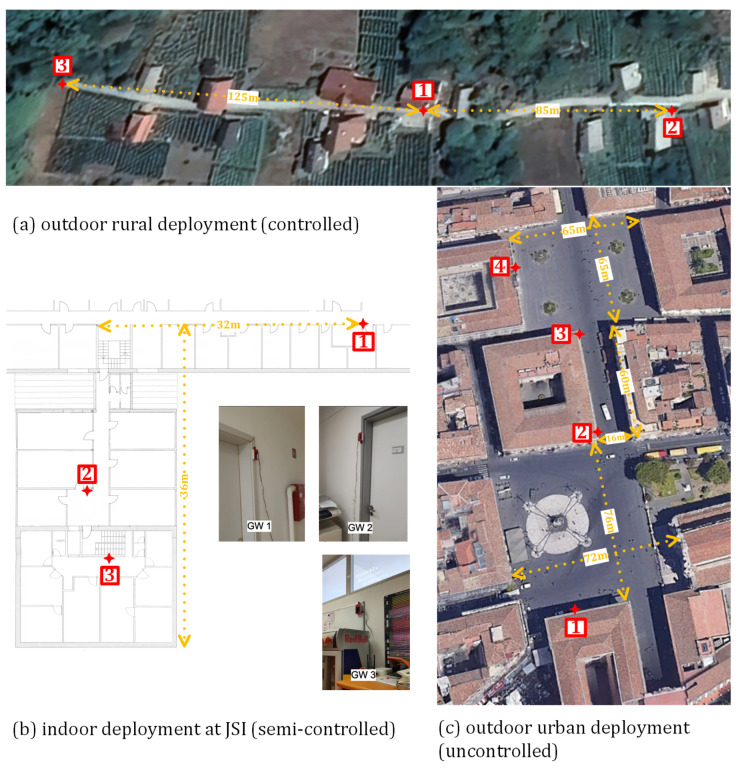
Locations of probe requests acquisitions.

For validation in a controlled rural environment, we deployed three WSDs for the acquisition of PRs, which were sending data to the remote database using a cellular network. As shown in Figure 6a, they were placed in three different locations with non-overlapping WiFi coverage. The three WSDs recorded no PRs when test devices were turned off; thus, the deployment can be characterized as completely controlled without external interference. Data gathered at this location are taken as a ground truth for each device individually and as group behavior when multiple devices were active near WSD.

A dataset for validation in a semi-controlled indoor environment was also obtained with three WSDs placed in the corridors of the Jozef Stefan Insitute, as depicted in Figure 6b. The WiFi coverage areas of the devices at locations 2 and 3 were partly overlapped, while the device at location 1 had no overlapping with other devices. In this case, data were sent to the collocated database via WiFi with a known global MAC address, which was subsequently filtered out of the dataset.

The last dataset was captured in an uncontrolled real-world environment with high-density pedestrian traffic in the city center of Catania, Italy. In this case, we deployed four WSDs on two main squares, Piazza del Duomo (locations 1 and 2) and Piazza Università (locations 3 and 4), which were connected with a busy pedestrian street Via Etnea, as shown in Figure 6c, and were collecting data over a period of several months. For sending data to the remote database, WSDs were connected to the Internet via ethernet or via WiFi access points with known global MAC addresses.

During the development and initial testing, the parameters of Algorithms A1 and A2 were first determined by observing PRs collected in an office environment and then fine-tuned using a publicly available labeled dataset [34]. Subsequently, the algorithms and the entire MAC de-randomization method were further validated in a controlled rural environment, where the results of the method were compared with ground truth, and in a semi-controlled indoor environment. Before starting the measurement campaign, we turned off all measurement devices and checked the environments for any remaining active WiFi devices. In the rural outdoor environment, we did not detect any unknown active device sending WiFi packets within the coverage areas of the deployed WSDs. In the indoor environment, the deployed WSDs detected several devices with active WiFi interfaces. These devices were excluded from the database used for validating the de-randomization method. In addition, to minimize the potential impact of uncontrolled WiFi-enabled devices carried by random people passing by, the measurement campaign was performed during the weekend.

After the verification of the environment, we started with data collection for individual devices. A brief summary of the devices used in measurement campaigns is given in Table 2. For each device, PRs generated within one minute were collected with the screen on, which was followed by PRs collected for one minute with the screen off. During these measurements, the other devices involved in the campaign were turned off in rural environment and had disabled WiFi interfaces in an indoor environment, so the recorded data for each device could later be used to determine the ground truth.

To test the MAC de-randomization method with three different levels of difficulty, the devices were divided into three groups. The first group comprised only devices from different manufacturers, so larger differences were expected between the PRs received from different devices. The second group contained only devices from one manufacturer (Samsung), which may aggravate the de-randomization process and thus distinguish unique devices. For the third group, a medium degree of difficulty in MAC de-randomization was expected, since half of the devices were from one manufacturer (Huawei) and the other half were from other different manufacturers. The distribution of devices among the groups is denoted in the last column in Table 2.

The same data collection procedure was applied for all three groups of devices in the rural and indoor environments at three different locations of WSDs indicated in Figure 6a,b. At each WSD location, PRs were collected from each group of devices for 10 min. Then, all three groups switched locations between WSDs, and the process was repeated. Thus, the database contains PR measurements from all three WSD locations at both measurement sites for all three groups of devices.

In the last testing scenario, all devices listed in Table 2 were grouped together. In the rural environment, data from received PRs were collected at one location for 10 min with screens on and for 10 min with screens off, whereas in the indoor environment, screens were on for 10 min. In the next step, all devices were moved to the location of the next WSD and PRs were measured for 10 min with screens off. In the rural outdoor scenario, the final step was to move the devices to location 2, where PRs were recorded for 10 min with the screens off.

The final testing and validation of the proposed MAC de-randomization method was conducted in a completely uncontrolled environment characterized by a much larger amount of PRs collected than the rural environment and indoor environments but with no ground truth. Thus, these tests did not focus on the accuracy of the proposed approach but rather on the robustness and scalability of the WSDs used to capture WiFi network management traffic, the remote database used to store the data, the PR pre-processing procedures and the MAC de-randomization method. Due to the large number of PRs collected, the statistical behavior of the proposed method can be better observed. In addition, such long-term data can also be exploited to identify daily/weekly/monthly patterns in user behavior, and combinations of WSDs can also be used to identify movement directions of identified WiFi-enabled devices.

The datasets used in this paper and the corresponding description are freely available to allow replication and continuation of the work. The dataset for a rural indoor environment is published in [37], while the dataset with all PRs collected by four WSDs for one week in the uncontrolled urban environment is published in [38].

### 5.2. MAC De-Randomization and Results Analysis

The results of the MAC de-randomization method for the labeled dataset are provided along with the list of devices in Table 3. In the first step, the proposed method was applied to the PRs of each device separately to determine the maximum differences between the PRs transmitted by the same device. In most cases, the method correctly identified a device. There were only a few cases where two devices were identified instead of one because (i) the PRs sent by the device were very different, (ii) the algorithm for matching global addresses with clusters of random addresses could not match a random MAC address with a global MAC address, and (iii) the device used two different types of addresses in PRs: one completely random and another with a fixed CID part. Since the devices sending two different addresses (random and with CID part) are rare (only one device in the labeled dataset used), this type of clustering is not considered as a separate case by the proposed method.

To further analyze the performance of the proposed method, the PRs of all 22 devices were aggregated. In this case, the proposed method correctly identified 21 devices (95.5%). This estimate of the total number of devices is a good approximation of the actual value, but we made a more detailed analysis of the clustered devices as allowed by the labeled dataset. It turned out that some of the 21 clusters formed by the MAC de-randomization contained PRs from more than one device. The method uniquely identified 11 devices (i.e., 11 clusters contained only one device with all its PRs). Two devices were fully identified by the random MAC addresses clustering algorithm, but they did not match or could not be matched with a global MAC address. The remaining clusters contained combinations of PRs from other devices due to some very similar PR’s IE data. Since the core of the MAC de-randomization method is the clustering of PRs with random MAC addresses, analyzing the clustering of only random addresses gives us a deeper insight into how the method works. When only random addresses were considered, the proposed method uniquely identified 10 out of 17 devices.

The final estimate of the total number of devices is still a good approximation of the actual value. If multiple devices send two different PRs, one of which contains only mandatory data that is very similar among the devices, then a cluster of multiple devices is formed based on these PRs, resulting only in one overcount. An undercount is caused by the similarity of PRs sent by similar devices (considering manufacturer and OS), so the proposed method has difficulty distinguishing between them. If the method is not able to match a global and a random MAC address of the same device, an undercount could be sometimes compensated by an overcount.

The validation results for the MAC de-randomization method in the rural and indoor environments are presented in Table 4, Table 5, Table 6 and Table 7. Similar as with the labeled dataset, the proposed method was first validated for data from each individual device separately for time intervals when the screen was on and off, which also revealed the differences in the behavior of the devices. In the rural environment, where no interfering devices were present, the proposed method successfully identified only one device for each of the devices tested. In the indoor semi-controlled environment, additional devices with global and random MAC addresses were also present during the data collection of individual tested devices. As explained in Section 5.1, these devices were identified and considered in the de-randomization. Thus, the final estimate of the de-randomization method includes the tested device and the additional uncontrolled devices whose PRs were detected during the corresponding time interval. The average ratio between the number of estimated devices and the actual number of devices was 96.7% . The main identified cause of errors was a mismatch between clustered devices with random MAC addresses and devices with global MAC addresses, while clustering only devices with random MAC addresses was error-free.

For further validation of the proposed method, three groups were formed according to Table 2. Table 5 shows the estimated values in the rural environment for each group for 10-min intervals at three different locations with the devices’ screen on. The mean values for identified devices in all scenarios were 91.7%, 87.5%, and 100% of the actual number of devices for the groups 1, 2, and 3, respectively, confirming the expectation that the distinction between devices of the same manufacturer is the most challenging. If considering only PRs with random MAC addresses detected during the data collection in the corresponding time interval, the proposed method detected 100%, 75%, and 100% of the actual devices. The source of error for the group 1 devices was an incorrect match between the device with the global MAC address and the cluster of devices with random MAC addresses. Since the group 2 devices consisted only of devices from the same manufacturer, the main error was due to the similar PRs of the devices, which caused the method to undercount the devices by one.

The results for the last scenario, where all devices are grouped together at three different time intervals, are summarized in Table 6. The proposed method detected on average 71.2% of actual devices. The percentage of the correctly identified devices decreases when the devices’ screens were turned off, since some devices did not send any PRs in this measurement period. If considering only PRs with random MAC addresses detected during the corresponding time interval, the method identified on average 67.5% of devices. This performance deterioration was caused by two sources of error, namely (i) false matches between a device with a global MAC address and a cluster of devices with random MAC addresses, and (ii) the similarity of PRs received from the same or similar devices that the proposed method could not distinguish.

In the indoor environment, the coverage areas of two WSDs were overlapping. Therefore, the proposed method was tested by each group of devices at the non-overlapping location and with the combinations of the two groups at the overlapping locations. Finally, the proposed method was validated with PRs sent by all devices merged into a single group. The results in Table 7 show that on average, the proposed algorithm identified 91.3% of the actual present devices. When only PRs with random MAC addresses were clustered, it recognized 83% of the devices. A more in-depth analysis of the results showed that the sources of errors were the same as in the rural environment.

The robustness and scalability of the proposed method was tested with the dataset collected by the WSD installed at Piazza del Duomo in Catania (location 1 in Figure 6c). The numbers of received PRs and identified WiFi-enabled devices are listed in Table 8. The proposed method was executed at hourly intervals over a 24 h period from midnight to midnight on 23 September 2022. The starting hour in the table corresponds to the local time of the beginning of the hourly interval. The number of PRs recorded in an hour and the PRs with actual unique data are shown to illustrate the amount of computation required by the proposed method. It should be noted that the number of PRs in such urban environment increases significantly compared to less populated environments, up to several hundreds per minute.

**Figure 7:**
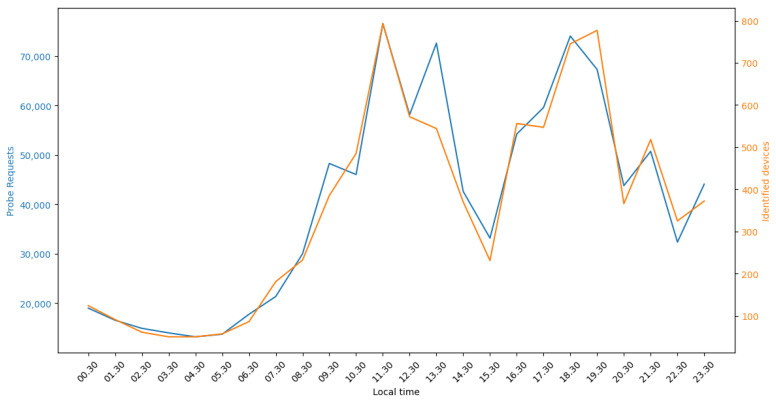
Hourly number of collected PRs and identified devices for one day at Piazza del Duomo, Catania.

The number of PRs and the number of devices identified by the proposed method in an hourly time slot for the 24 h period are also shown in Figure 7. It can be observed that the number of PRs coincides very well with the number of identified devices. During night-time hours, a higher number of devices than expected are identified due to nearby static devices not being filtered out. Since the method compares each PR with all the remaining PRs, the computational complexity of the method increases exponentially with the number of recorded PRs. Therefore, in busy urban environments with a high volume of pedestrians and consequently a huge number of recorded PRs, the identification of unique devices may not be possible in real time, indicating one possible direction for further optimization of the algorithm.

### 5.3. Discussion

Extensive validation of the proposed method using datasets from controlled rural, semi-controlled indoor, and uncontrolled urban environments confirms its ability to accurately detect unique active WiFi interfaces.

Comparison of the results with existing work applying similar approaches is difficult since their validation was performed in public places by counting the number of people and without the information about the number of WiFi-enabled devices carried by the individual (e.g., tickets sold [31]), so no ground truth can be determined. An approximate comparison can be made with the solutions where validations were performed with measurements that includes the information on the number of WiFi-enabled devices as a ground truth. In [11], the accuracy of 91.3% was achieved in the laboratory environment, while in [32], 96% accuracy in the laboratory environment and 75% in a real-world scenario is reported. With the publicly available dataset [34], we obtained similar results of 95.5%, while in the controlled environment, we achieved accuracy of at least 87.5% for smaller groups of devices. In rural environments the achieved accuracy was over 70%, and in indoor environments for larger groups, it was 84%.

A more accurate comparison can be made with the findings reported in [32,33], where the authors used the same labeled, publicly available dataset as in this work [34]. Although the results in [32] are not explicit, since only the behavior of the proposed method was tested under different parameters, they claim to achieve an accuracy of up to 95%, which is similar to the accuracy achieved with our proposed method. In [33], the authors achieved an accuracy of 92% and also performed a more thorough analysis of the homogeneity of the formed clusters, achieving values of up to 0.97. High homogeneity values can be obtained in the case where devices grouped in clusters send different number of PRs. From the paper, it is not clear whether they filtered PRs with the same data and thus considered only unique PRs of devices. Related works focused only on the de-randomization of random MAC addresses, while we considered the real-world scenario that includes some special cases, such as devices transmitting both random and global MAC addresses that need to be matched to avoid overcounting the devices.

In general, the results show that the proposed method tends to undercount the devices, which is mainly due to the similarity of PRs of the same or similar devices. The method performs well for small groups of devices, while for larger groups, the differences between PRs become less significant, resulting in an underestimate of the number of identified devices. This effect could be reduced by fine-tuning the model parameters depending on the scenario. In addition, the scaling test with an extremely large urban dataset revealed some drawbacks in terms of exponential computational complexity. Thus, the two main drawbacks of the proposed de-randomization method based on PR’s IE values are (i) the need to configure multiple parameters, which also can be seen as an advantage, as it allows adapting the method to the specificites of the environment or application, and (ii) the computational effort required to compare the PRs of a single device with all the PRs of other devices. On the other hand, the proposed approach has several advantages. It is a low-cost solution that requires only WSDs operating in the monitor mode and remote data storage capabilities, the system is easy to set up, and it is completely unobtrusive to people, since no particular actions are required from the owners of the WiFi-enabled devices. The system not only estimates the number of people in close proximity to the WSD but can also provide information on clustered data that can be used to analyze the movements of the crowd and individuals.

Validation of the proposed method showed that many of the tested devices which use random MAC addresses are changing them very rarely. Therefore, the approach could be further improved by identifying these devices based on the number of PRs detected. When comparing PRs sent by devices that change the MAC address at every burst and PRs sent by devices that change it infrequently, a large difference in the number of PRs per address was observed, which can be used to set a higher threshold for matching these two types of devices. This would also reduce the errors caused by incorrectly matching a global MAC address with a clusters of random MAC addresses.

Similarly, the observation of a high correlation between the number of collected PRs and the number of identified devices, as seen in Figure 7, could lead to a substantial simplification of the method in which the number of devices could be estimated from the number of PRs recorded. Further data collection would be required to determine whether the correlation is indeed strong enough. Such a simplification would only be applicable in areas where there is a large number of devices so that the statistics becomes sufficiently robust. As manufacturers change the frequency of sending PRs, the simplified approach would also need to be re-evaluated over time. However, simplifying the method in this way would also preclude the ability to use the same data obtained from PRs for monitoring the movement between the coverage areas of neighboring WSDs.

Fine-tuning the parameters of the proposed method proved to be time-consuming and not optimized. Therefore, a procedure to automatically determine and optimize the coefficients would have to be implemented. A more detailed analysis of the importance of each bit of the IE fields could also be performed to include only the most important bits to distinguish between devices. In addition, other features could be extracted from ToA, such as the inter-frame and inter-burst times.

The exponential complexity of the proposed method is another challenge for future optimization if it is expected to run for longer periods of time or for multiple locations. The results presented in this work were calculated on a consumer-level hardware, but further method optimization should be considered if more data are to be processed or it should be adapted for the use on high-performance computing infrastructure.

## 6. Conclusions

This paper presented an approach for monitoring the presence of individuals at specific locations based on collected PRs, taking into account the increasing adoption of MAC address randomization due to privacy concerns. The main contribution of this work is the development of a method for MAC de-randomization based on the similarity of PRs, more specifically IE data. We described the designed system and deployment for capturing PRs sent by WiFi-enabled devices. The detection of unique WiFi interfaces is implemented in two stages. After grouping PRs based on random or global MAC addresses, clustering is performed on PRs from devices with random MAC addresses. The generated clusters are then matched with PRs of devices with global MAC addresses.

The proposed approach was validated in a controlled rural, semi-controlled indoor, and uncontrolled urban environment, first with PRs from only individual WiFi-enabled devices and later with all devices and three formed groups of devices to account for three different levels of difficulty for de-randomization. Validation on individual devices showed that in some cases, the de-randomization method detected two devices instead of one. For the formed groups, the results show the disadvantage of the proposed method when multiple devices of the same manufacturer and version of OS were present. Although the performance of the proposed method decreases in this case, it is still above 70% for rural and 80% for indoor environments.

In the paper, we identified some potential improvements and shortcomings of the proposed methods that could be addressed as part of future work such as the automatic fine-tuning of parameters, reduction of computational complexity, optimization of the threshold for matching devices with large differences in the number of detected PRs, and extraction of additional features from ToA. Another challenge for future work is to extend the proposed approach of detecting unique WiFi-enabled devices to a system that analyzes the collected data from multiple locations in the urban area to determine the movement patterns of users and use these data for traffic management, route optimization, resilience planning, etc.

## Figures and Tables

**Table 1:** Selected IEs, their data type and data length.

IE Name	Data Type	Data Length in Octets
SSID	UTF-8 encoded	Variable (max 32)
Supported Data Rates	Each data rate encoded as one octet	Variable (max 8)
Extended Supported Rates	Each data rate encoded as one octet	Variable (max 255)
HT Capabilities	Hex	26
Extended Capabilities	Hex	Variable
Interworking	Hex	1–9
VHT Capabilities	Hex	12
Vendor Specific Tag	Hex	Variable
Extended Tag	Hex	Variable

**Table 2:** Summary of devices used for the acquisition of PRs.

Device Name	OS	MAC Type	Assigned Group
Apple iPhone 12 Pro	iOS 16	Random MAC only	1
Nokia 7 Plus	Android 10	Random MAC only(CID: da:a1:19)	1
Samsung S10E	Android 12	Random MAC only	1
Samsung J3 2016	Android 5.1.1	Global MAC only (d0:b1:28:d2:de:e5)	2
Samsung S3	Android 4.4.4	Global MAC only (34:23:ba:d5:34:1b)	2
Samung Galaxy Nexus	Android 4.3	Global MAC only (a0:0b:ba:da:64:7e)	2
Samsung S10E	Android 12	Random MAC only	2
Samsung S7 Edge	Android 8	Random MAC only	2
Samsung J5	Android 6	Global MAC only (20:55:31:fc:4c:86)	2
Samsung S7	Android 8	Random MAC only	2
Samsung S7	Android 8	Random MAC only	3
Samsung Tab S8	Android 12	Random MAC only	2
Huawei Nexus 6P	Android 8.1.0	Global MAC (dc:ee:06:fd:8c:9a) + Random MAC (CID: da:a1:19)	3
Huawei P20	Android 10	Global MAC (e4:34:93:b5:f0:74) + Random MAC (CID: da:a1:19)	3
Huawei P20	Android 10	Global MAC (e4:0e:ee:3e:3e:44) + Random MAC (CID: da:a1:19)	3
Huawei P30 Lite	Android 10	Random MAC only (CID: da:a1:19)	1
Huawei P20 Lite	Android 9	Random MAC only (CID: da:a1:19)	3
Asus Tab 8"	Android 5.0	Global MAC only (54:a0:50:0e:8f:ee)	1
Asus Tab 7"	Android 4.2.2	Global MAC only (08:62:66:72:ac:1f)	3
OnePlus 3	Android 9	Random MAC only (CID: da:a1:19)	3
OnePlus 6	Android 11	Global MAC only (64:a2:f9:28:98:6c)	1
Lenovo VIBE A7020	Android 6	Global MAC only (54:27:58:30:ac:5a)	1
Xiaomi Poco F1	Android 10	Random MAC only	1

**Table 3:** MAC de-randomization results for individual devices from the labeled dataset.

Device	Global Addresses Detected	Random Addresses Detected	Devices Identified
Samsung Galaxy M31	0	15	1
Xiaomi Redmi 4	0	531	2
Samsung Galaxy S4	1	0	1
Huawei ALE-L21	1	0	1
Xiaomi Mi A2 Lite	0	435	2
Huawei CLT-L09 (P20)	1	0	1
Samsung Galaxy S6 edge (SM-G928F)	1	0	1
Samsung Galaxy S7	0	38	1
Xiaomi Redmi 5 Plus	0	253	2
Samsung Galaxy J6	1	26	2
Google Pixel 3A	0	46	2
Apple XS max	0	103	1
Apple iPhone 6	0	57	1
One Plus Nord	0	35	1
Huawei VTR-L09 (P10)	1	0	1
Huawei STF-L09 (Honor 9)	1	88	1
Xiaomi Redmi Note 7	1	153	1
Xiaomi Redmi Note 9S	0	138	1
Apple iPhone XR	0	36	1
Google Pixel 3A	0	23	1
Apple iPhone 12	0	1206	1
Apple iPhone 7	0	19	1
All devices combined	8	3201	21/22 (95.5%)

**Table 4:** MAC de-randomization results for individual devices for the rural and indoor environments.

		Global Addresses Detected		Random Addresses Detected		Devices Identified/Devices Present	
	**Location**	**Rural**	**Indoor**	**Rural**	**Indoor**	**Rural**	**Indoor**
** Device**							
Apple iPhone 12 Pro		0	6	31	19	1/1	8/8
Nokia 7 Plus		0	6	6	20	1/1	8/8
Samsung S10e		0	5	6	15	1/1	8/9
Samsung J3 2016		1	7	0	7	1/1	8/8
Samsung S3		1	7	0	6	1/1	9/9
Samsung Galaxy Nexus		1	6	0	10	1/1	8/9
Samsung S7		0	5	4	22	1/1	9/9
Huawei Nexus 6P		1	6	2	14	1/1	8/8
Asus Tab 8"		1	6	0	10	1/1	7/7
Asus Tab 7"		1	6	0	11	1/1	7/7
OnePlus 3		0	5	1	16	1/1	7/9
Samsung S10e		0	8	4	4	1/1	9/9
Samsung S7 Edge		0	8	4	4	1/1	9/9
Samsung J5		1	8	0	0	1/1	8/8
Samsung S7		0	8	3	1	1/1	9/9
Samsung Tab S8		0	9	4	12	1/1	12/12
Huawei P20		1	8	3	0	1/1	8/8
Huawei P20			8		3		8/8
Huawei P30 Lite		0	8	1	1	1/1	8/9
Huawei P20 Lite		0	8	1	1	1/1	8/9
OnePlus 6		1	10	0	0	1/1	10/10
Lenovo VIBE A7020		1	8	0	7	1/1	9/10
Xiaomi Poco F1		0	7	2-5	16	1/1	10/10
					**Mean**	**100%**	**96.7 %**

**Table 5:** MAC de-randomization results for groups of devices for the rural environment.

	Global Addresses Detected			Random Addresses Detected			Devices Identified			Devices Identified		
										(Only Random MACs)		
**Loc.**	**Group**			**Group**			**Group**			**Group**		
	**1**	**2**	**3**	**1**	**2**	**3**	**1**	**2**	**3**	**1**	**2**	**3**
**Rural 1**	3	4	3	50	16	6	7/8	7/8	6/6	5/5	3/4	4/4
**Rural 3**	3	4	3	92	17	7	8/8	7/8	6/6	5/5	3/4	3/3
**Rural 2**	3	4	3	54	25	5	7/8	7/8	6/6	5/5	3/4	3/3
					**Mean**		**91.7%**	**87.5%**	**100%**	**100%**	**75%**	**100%**

**Table 6:** MAC de-randomization results for all devices in a single group for the rural environment.

Loc./Scenario	Global Addresses Detected	Random Addresses Detected	Devices Identified	Devices Identified (Only Random MACs)
**Rural 2/screen on**	10	92	17/22	8/12
**Rural 2/screen off**	8	97	14/22	8/12
**Rural 2/screen on + screen off**	10	188	16/22	9/13
		**Mean**	**71.2 %**	**67.5 %**

**Table 7:** MAC de-randomization results for groups of devices for the indoor environment.

Group/Loc	Global Addresses Detected	Random Addresses Detected	Devices Identified	Devices Identified (Only Random MACs)
**Group 1/1**	8	63	13/13	6/6
**Group 2/1**	9	11	12/13	3/4
**Group 3/1**	9	10	12/12	4/5
**Groups 1,3/2,3**	18	54	24/26	7/8
**Groups 2,3/2,3**	19	47	25/26	6/7
**Groups 1,2/2,3**	16	57	22/25	7/9
**All devices/3**	20	105	27/32	9/12
		**Mean**	**91.3 %**	**83 %**

**Table 8:** MAC de-randomization results for one day at Piazza del Duomo, Catania.

Start of Hourly Interval	All PRs/ Unique PRs	Global Addresses Detected	Random Addresses Detected	Devices Identified
00:00:00	18,980/5637	82	3112	124
01:00:00	16,531/4448	59	2055	91
02:00:00	14,895/3985	37	1657	61
03:00:00	13,973/3115	28	802	50
04:00:00	13,174/2655	24	598	50
05:00:00	13,700/2716	32	609	57
06:00:00	17,736/3902	50	1688	86
07:00:00	21,342/6141	124	3044	181
08:00:00	29,980/10,918	166	8121	232
09:00:00	48,273/21,079	237	17,606	385
10:00:00	46,029/21,150	347	17,225	485
11:00:00	76,586/38,601	520	32,869	793
12:00:00	58,161/26,319	401	22,234	572
13:00:00	72,632/35,171	305	30,214	544
14:00:00	42,608/20,848	257	17,938	370
15:00:00	33,156/14,899	160	12,673	231
16:00:00	54,233/25,086	405	20,621	556
17:00:00	59,599/28,587	356	23,853	547
18:00:00	74,070/33,673	508	27,763	745
19:00:00	67,298/31,440	592	25,821	777
20:00:00	43,776/20,274	254	16,735	366
21:00:00	50,731/23,810	409	19,629	518
22:00:00	32,345/15,197	228	12,855	325
23:00:00	44,087/21,058	259	17,792	372

## Data Availability

The data presented in this study are openly available in repositories Zenodo [37,38] and Mendeley Data at https://doi.org/10.17632/j64btzdsdy.1.

## Abbreviations

The following abbreviations are used in this manuscript:

CID	Company Identifier
GDPR	General Data Protection Regulation
HMM	Hidden Markov Models
HT	High Throughput
IE	Information Element
IFAT	Inter-Frame Arrival Time
IoT	Internet-of-Things
JSI	Jozef Stefan Institute
JSON	JavaScript Object Notation
MAC	medium access control
ML	Machine learning
NIC	Network Interface Controller
OPTICS	Ordering Points to Identify the Clustering Structure
OS	Operating system
OUI	Organization Unique Identifier
PR	Probe Request
REST	Representational State Transfer
rPi	Raspberry Pi
RSSI	Received Signal Strength Indicator
SSID	Service Set Identifier
ToA	Time of Arrival
VHT	Very High Throughput
WSD	Wireless Sensor Device

## Appendix A. Structure of Stored Data from PR

Data from PRs of each MAC address are stored in the following JSON format:

## Appendix B. Algorithms

**Algorithm A1** Algorithm for calculating distance between two probe requests
**procedure**dist_btwn_two_probe_req($PR\text{\_}IE\text{\_}data_{1},SSIDs_{1}PR\text{\_}IE\text{\_}data_{2},SSIDs_{2}$) distance←STARTING_DISTANCE **for each** IE_key $PR\text{\_}IE\text{\_}data_{1}$ and $PR\text{\_}IE\text{\_}data_{2}$ have in common **do** **if** IE_key **equal** DATA_RTS **then** **merge** Supported Data Rates and Extended Data Rates for both PRs **increase** distance for each data rate of symetric difference multiplied by IE’s specific scale **if** IE_key **equal** HT_CAP **Or** VHT_CAP **then** $distance\leftarrow distance+\frac{number\text{\_}of\text{\_}distinguishing\text{\_}bits}{number\text{\_}of\text{\_}all\text{\_}bits}*specific\text{\_}IE\text{\_}scale$ **if** IE_key **equal** EXT_CAP **Or** INTERWORKING **then** **if** $length(probe\text{\_}req\text{\_}data_{1}\left[IE\text{\_}key\right]!=length(probe\text{\_}req\text{\_}data_{2}\left[IE\text{\_}key\right]$ **then** distance←distance+specific_IE_value **else** $distance\leftarrow distance+\frac{number\text{\_}of\text{\_}distinguishing\text{\_}bits}{number\text{\_}of\text{\_}all\text{\_}bits}*specific\text{\_}IE\text{\_}scale$ **if** IE_key **equal** VENDOR_SPEC **then** distance←distance−special_IE_koef **increase** distance for vendor ID of symetric difference multiplied by IE’s specific scale **decrease** distance for each intersecting vendor ID multiplied by IE’s specific scale **for each** intersecting vendor ID **do** **decrease** distance for each intersecting data ID multiplied by IE’s specific scale **for each** intersecting data ID **do** $distance\leftarrow distance+\frac{number\text{\_}of\text{\_}distinguishing\text{\_}bits}{number\text{\_}of\text{\_}all\text{\_}bits}*specific\text{\_}IE\text{\_}scale$ **if** IE_key **equal** EXT_TAG **then** distance←distance−special_IE_koef **increase** distance for each data ID of symetric difference multiplied by IE’s specific scale **decrease** distance for each intersecting data ID multiplied by IE’s specific scale **for each** intersecting data ID **do** $distance\leftarrow distance+\frac{number\text{\_}of\text{\_}distinguishing\text{\_}bits}{number\text{\_}of\text{\_}all\text{\_}bits}*specific\text{\_}IE\text{\_}scale$ **decrease** distance for intersecting SSID multiplied by IE’s specific scale **increase** distance by number of IEs of symetric difference relative to number of intersecting IEs multiplied by specific scale tmp_dst←Inf **for each** $RSSI_{1},TOA_{1}$ in $probe\text{\_}req\text{\_}data_{1}$ **do** **for each** $RSSI_{2},TOA_{2}$ in $probe\text{\_}req\text{\_}data_{2}$ **do** $dst\text{\_}RSSI\leftarrow RSSI\text{\_}KOEF^{|RSSI_{2}-RSSI_{1}|}-1$ dst_TOA←0 **if** $|TOA_{2}-TOA_{1}|<TIMING\text{\_}THRESHOLD$ **then** dst_TOA←TIMING_KOEF **if** (dst_RSSI+dst_TOA)<tmp_dst **then** tmp_dst←dst_RSSI+dst_TOA distance←distance+tmp_dst **if** distance<MINIMUM_DISTANCE **then** distance←MINIMUM_DISTANCE **return** distance

**Algorithm A2** Reachability distance-based clustering
**procedure**Clustering(reach_dists,xi_up,xi_down,epsMax) in_cluster←False labels[:]←−1 current_label←0 climbing←False falling←False **for** i←1 to length(reach_distss)−1 **do** **if** in_cluster **then** **if** reach_dists[i+1]>reach_dists[i] **then** **if** not climbing **then** climbing←True value_start_climbing←reach_dists[i] **if** $\frac{reach\text{\_}dists[i+1]}{value\text{\_}start\text{\_}climbing}>xi\text{\_}up$**Or**reach_dists[i+1]>epsMax **then** in_cluster←False current_label←current_label+1 climbing←False **else** labels[i+1]←current_label **else** climbing←False labels[i+1]←curr_label **else if** reach_dists[i+1]<reach_dists[i] **then** **if** not falling **then** value_start_falling←reach_dists[i] index_start_falling←i falling←True **if** reach_dists[i+1]<epsMax And $\frac{reach\text{\_}dists[i+1]}{value\text{\_}start\text{\_}falling}<xi\text{\_}down$ **then** labels[index_start_falling−1:i+1]←curr_label in_cluster←True falling←False **else** falling←False i←i+1 **return** labels
